# Detecting cell-in-cell structures in human tumor samples by E-cadherin/CD68/CD45 triple staining

**DOI:** 10.18632/oncotarget.4275

**Published:** 2015-06-18

**Authors:** Hongyan Huang, Ang Chen, Ting Wang, Manna Wang, Xiangkai Ning, Meifang He, Yazhuo Hu, Long Yuan, Shichong Li, Qiwei Wang, Hong Liu, Zhaolie Chen, Jun Ren, Qiang Sun

**Affiliations:** ^1^ Department of Oncology, Beijing Shijitan Hospital of Capital Medical University, Beijing, P. R. China; ^2^ Laboratory of Cell Engineering, Institute of Biotechnology, Beijing, P. R. China; ^3^ Department of Vascular and Endocrine Surgery, Xijing Hospital, Fourth Military Medical University, Xi'an, China, Xi'an, Shaanxi Province, P. R. China; ^4^ Beijing Key Laboratory for Aging and Geriatrics, Institute of Geriatrics, General Hospital of Chinese PLA, Beijing, P.R.China

**Keywords:** cell-in-cell structures, cell cannibalism, EML method, epithelium, macrophage

## Abstract

Although Cell-in-cell structures (CICs) had been documented in human tumors for decades, it is unclear what types of CICs were formed largely due to low resolution of traditional way such as H&E staining. In this work, we employed immunofluorescent method to stain a panel of human tumor samples simultaneously with antibodies against E-cadherin for Epithelium, CD68 for Macrophage and CD45 for Leukocytes, which we termed as “EML method” based on the cells detected. Detail analysis revealed four types of CICs, with tumor cells or macrophage engulfing tumor cells or leukocytes respectively. Interestingly, tumor cells seem to be dominant over macrophage (93% vs 7%) as the engulfer cells in all CICs detected, whereas the overall amount of internalized tumor cells is comparable to that of internalized CD45^+^ leukocytes (57% vs 43%). The CICs profiles vary from tumor to tumor, which may indicate different malignant stages and/or inflammatory conditions. Given the potential impacts different types of CICs might have on tumor growth, we therefore recommend EML analysis of tumor samples to clarify the correlation of CICs subtypes with clinical prognosis in future researches.

## INTRODUCTION

Cell-in-cell structures (CICs) refer to the unusual structures with one or more viable cells transiently existing inside other one. Early reports on CICs could be dated back more than a century to at least the middle of the 19th century, when Eberth found some lymphocytes enclosed inside intestinal epithelial cells [1]. Later on throughout 20^th^ century, similar structures were frequently observed in a variety of human specimens, with tumors the most documented tissues. Interestingly, sporadic evidences from various types of tumor tissues supported a positive correlation of CICs with malignant tumors, that is, more CICs in higher tumor grades [2–6]. Accordingly, CICs index was proposed to assistant pathological evaluation of tumor malignancy [2, 4, 7].

The tight correlation of CICs with human tumors suggested its functional implications in tumor development and progression, consistent with which, CICs' roles in human tumors, although still in debate, are being revealed. Recent studies indicated that CICs formation could lead to the death of internalized cells, thus would limit tumor growth by engulfing and killing tumor cells [8]. Indeed, works from us and other groups showed that tumor growth was drastically inhibited upon CICs induction in tumor cells [9, 10], or vice versa, supporting a tumor suppressive role of CICs. Nevertheless, CICs formation may promote tumor survival and progression by consuming ingested cells [11–13] or inducing aneuploidy [14, 15]. Lately, CICs formation was demonstrated to be a mechanism of cell competition in mammals, which functions to promote tumor evolution by selecting malignant cells with oncogenic mutations conferring lower Rho activity [16–18].

It is now clear that CICs could be formed homotypically among tumor cells [9, 10, 19], or heterotypically between tumor cells and some leukocytes, such as natural killer (NK) cell, T and B lymphocytes [12, 14, 20]. In addition, macrophage could also target live tumor cells for engulfment to generate CICs [21, 22], which are morphologically indistinguishable from engulfer of tumor cells. Despite of the complex CICs subtypes existing in human tumor tissues, previous reports on CICs rarely analyzed the identities of engulfers and/or internalized cells probably due to low resolution of traditional staining methods like H&E and Giemsa, which might be responsible for the controversial correlation of CICs with pathological scoring [2, 3, 23–25]. In light of this, we attempted to analyze the CICs subtypes in human tumors by using immunofluorescent staining. Tumor sections were co-stained with antibodies against E-cadherin, CD68 and CD45 to label epithelial tumor cells, macrophages and leukocytes respectively. For high throughput analysis, the multispectral imaging and analysis system (Vectra-Nuance-InForm®) from *Perkin Elmer* were employed to analyze the tumor sections labeled with multiple fluorophores. As a result, four subtypes of CICs were indentified in human tumor samples from seven tissues, including breast gland, liver, colon, stomach, prostate, pancreas and lung. Since each subtype of CICs is formed through distinct mechanisms and might implicate different cellular outcomes, we therefore proposed that it might be necessary to conduct detail subtype analysis before making a clinical relevance for CICs in human tumors.

## RESULTS

### E-cadherin/CD68/CD45 triple staining of human tumor TMA

**Figure 1:**
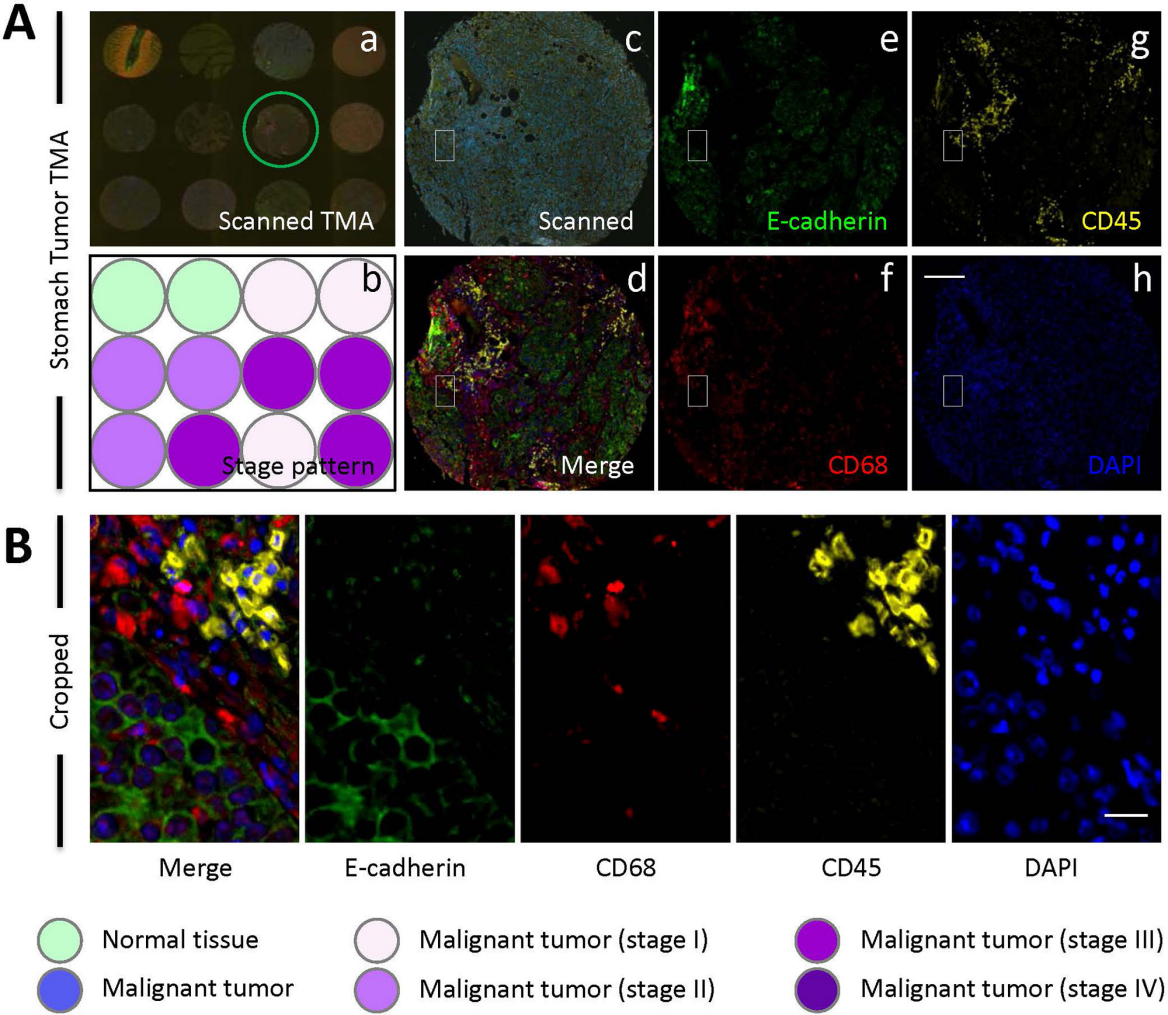
E-cadherin/CD68/CD45 triple staining of stomach TMA **A.** Images of human stomach TMA stained with antibodies for E-cadherin, CD68 and CD45. Nuclei were counterstained with DAPI. Left panel shows a stitched image of whole TMA with multiple fluorescent channels merged (a) and schematic illustration of tumor stage of each core (b); Middle panel shows representative core image of the TMA circled in (a), with the upper one (c) showing the raw scanned image and lower one (d) showing spectral-processed image of (c). The right panels (e-h) display images of four unmixed single fluorescent channels as indicated. Scale bar: 150 μm. **B.** Left image shows cropped region of core image (A-d), right images show four unmixed single fluorescent channels as indicated. Scale bar: 20 μm.

**Figure 2:**
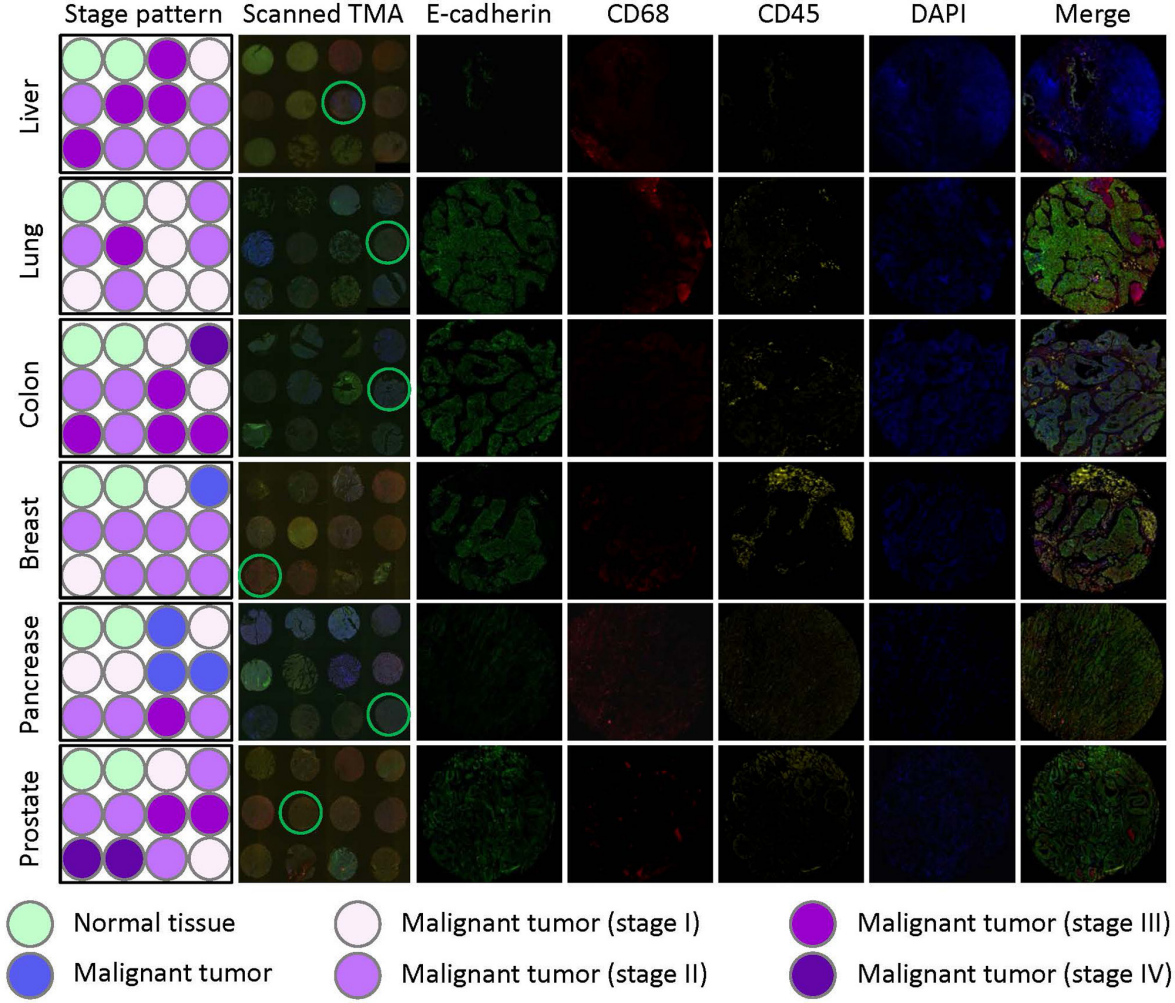
E-cadherin/CD68/CD45 triple staining of multiple TMAs Multiple human tumor TMAs were stained with antibodies for E-cadherin, CD68 and CD45. Nuclei were counterstained with DAPI. Left two panels show the schematic illustrations of tumor stage of each core (stage pattern) and stitched images of whole TMA (scanned TMA), respectively. Right five panels show unmixed images of single fluorescent channels and merged composite images for the cores circled in scanned TMA panel. TMAs from tumors of liver, lung, colon, breast, pancreas and prostate were stained and displayed.

As introduced above, three types of cells might participate in CICs formation, including tumor cells, macrophages and leukocytes. To differentiate these cell types in tumor tissues, we chose marker antibodies for each cell type. E-cadherin antibody was chosen to label cancerous tumor cells, which also posses the advantage to display cell boundaries as E-cadherin is a transmembrane protein. CD68 antibody was used to label macrophages, and CD45 for leukocytes. For each marker, we tested antibodies from different companies and species so that they are compatible with paraffin-embedded sections and multispectral staining. As a result, we obtained one antibody for each marker which is suitable for staining paraffin-embedded sections. However, we failed to have them compatible with simultaneous multispectral staining by traditional method as two (antibodies for E-cadherin and CD45) of them are from the same species (mouse). To solve this problem, we introduce into our staining the Opal Multiplex tissue staining method (*Perkin Elmer*), which allows multispectral staining irrespective of antibody species. Since we currently have no clues on in which tumor types and what types of tumors CICs could be detected, we used commercially available tumor microarray, where multiple tumor samples were plotted on one slide, for the staining. The stained slides were then mounted for imaging, spectral unmixing and analysis by the multispectral imaging and analysis system (Vectra-Nuance-InForm®) from *Perkin Elmer*. As shown in Figure 1A, the multispectral images stained by this method could be successfully unmixed into four spectral channels (DAPI, FITC, Cy3 and Cy5), the unique distribution pattern of each fluorescent channel was consistent with separate localization of each cell type within the stomach tumor tissues. As expected, epithelial tumor cells were positive in E-cadherin, while CD68^+^ macrophages and CD45^+^ lymphocytes were present abundantly in the inter-epithelium matrix with some infiltrated in between (Figure 1B). Staining was also performed in TMA for human tumors from other organs including liver, lung, colon, breast gland, pancreas and prostate, specific and unmixable signals were obtained for all of them (Figure 2). That the three types of cells, as evidenced by different staining pattern of each antibody, could be readily and specifically detected in a range of tumor tissues suggests that the method we employed works well in our experiments.

### Presence of CICs in multiple human tumors

**Figure 3:**
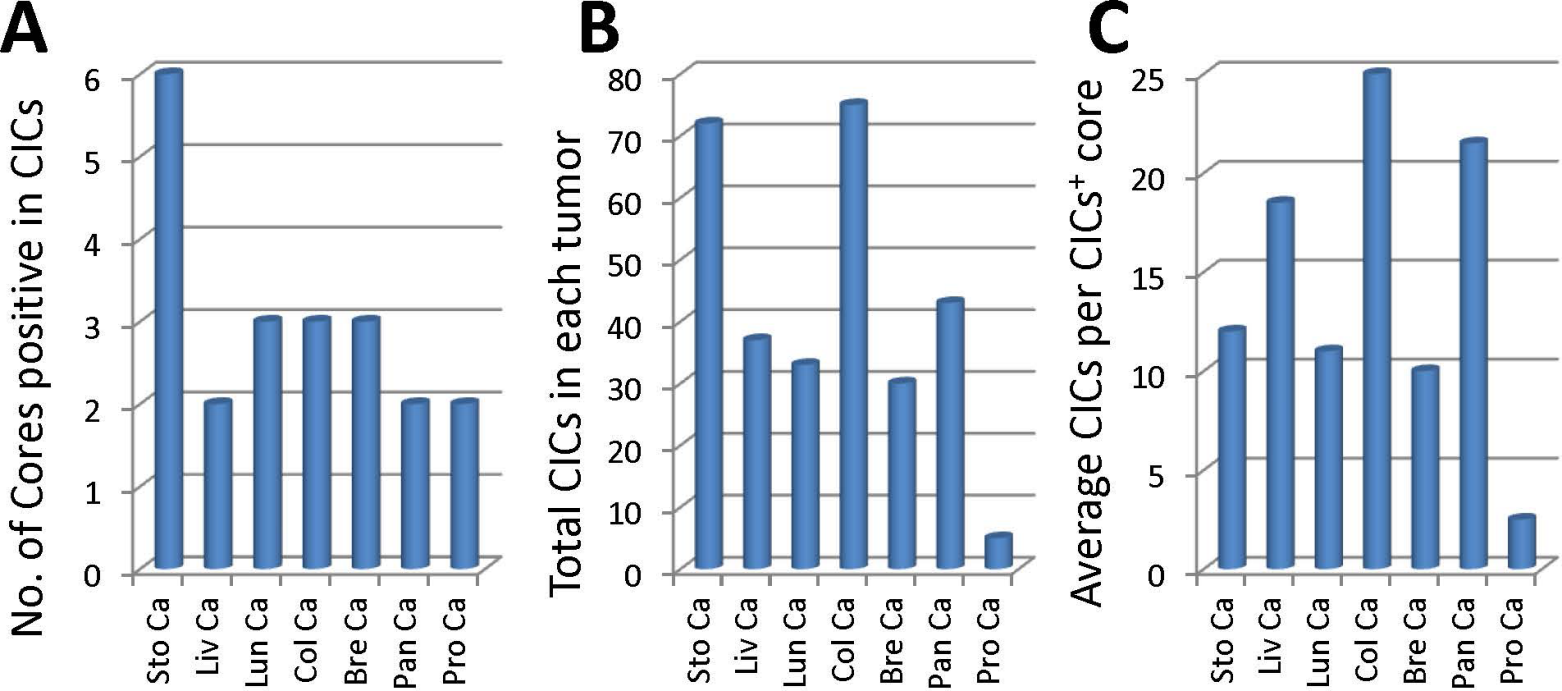
Profile of CICs detected in various tumor samples **A.** Graph shows number of cores where CICs were detected in each tumor TMA. **B.** Quantification of total CICs detected in each tumor TMA. **C.** Average CICs number in each CICs-positive core for each tumor TMA. Sto Ca: stomach carcinoma; Liv Ca: liver carcinoma; Lun Ca: lung carcinoma; Col Ca: colon carcinoma; Bre Ca: breast carcinoma; Pan Ca: pancreas carcinoma; Pro Ca: prostate carcinoma.

We screened the presence of CICs in the stained tumor tissues from different organs. Only those structures with inner cells morphologically fully enclosed were counted. CICs seemed to be easier to be detected in stomach tumors, where 6 out of 10 cores were positive in CICs. For the rest of tumor types, the rates of CICs positivity were 2 or 3 out of 10 cores (Figure 3A). Accordingly, more CICs were indentified in stomach tumor samples, with which colon tumors had comparable number of CICs although only three cores were positive in CICs. Prostate tumors seemed to be resistant to CICs formation as only three CICs were identified in all 10 cores (Figure 3B). Meanwhile, tumors from colon and pancreas displayed more CICs in the CICs-positive cores (Figure 3C), suggesting that CICs might be formed in high frequency in tumors of specified clinical features, which warrant further analysis with enlarged sample number.

### Four subtypes of CICs in human tumor tissues

**Figure 4:**
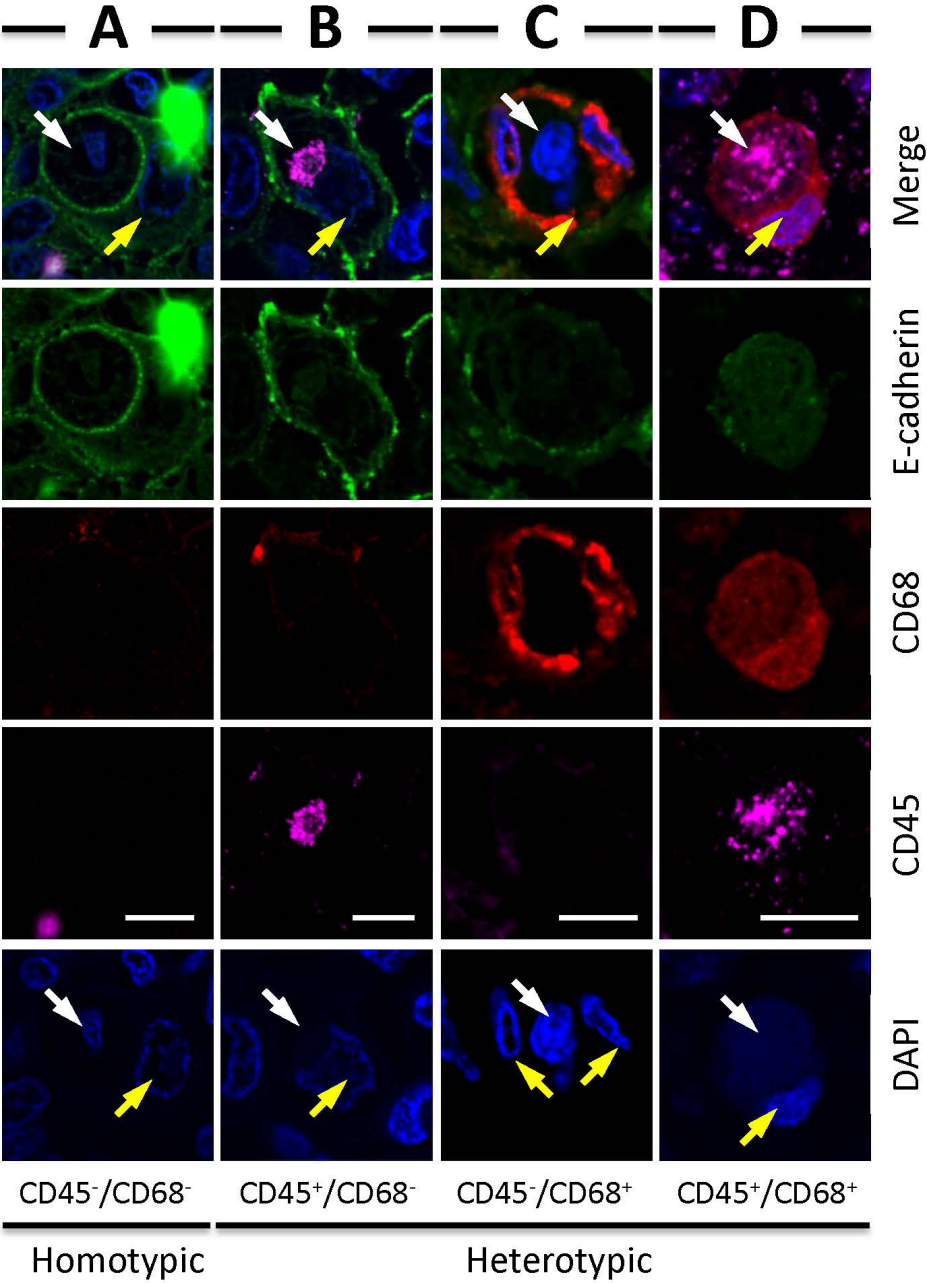
Four types of CICs detected in human tumors Representative images for each CICs subtype: CD45^−^/CD68^−^ **A.** CD45^+^/CD68^−^ **B.** CD45^−^/CD68^+^ **C.** CD45^+^/CD68^+^ **D.** White arrows indicate inner cells or their nuclei, yellow arrows indicate outer cells or their nuclei. Scale bar: 20 μm.

**Figure 5:**
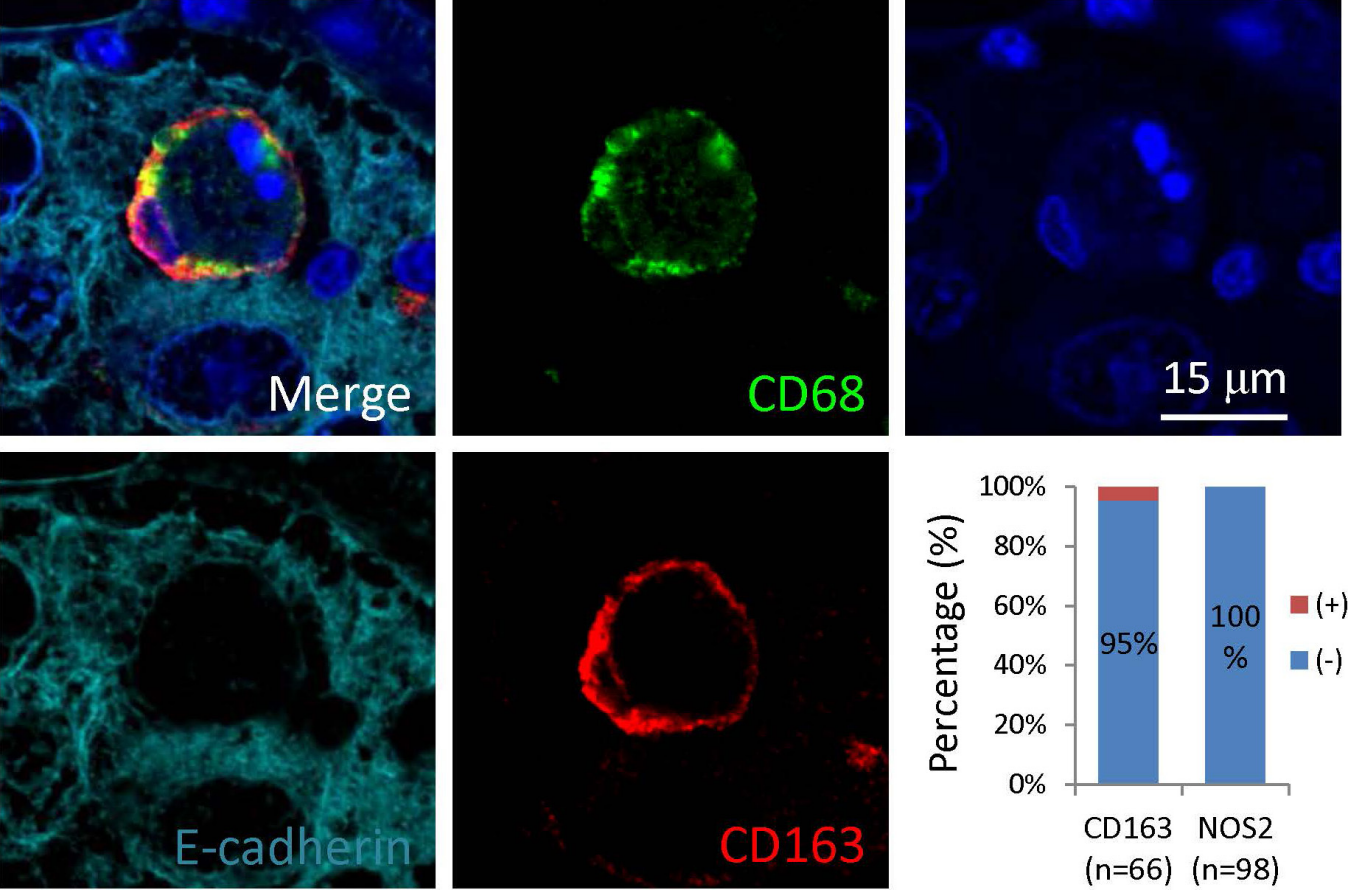
Macrophages positive in CD163 participate in CICs formation in human tumors Representative image is displayed in merged or single pseudo-color channel. Graph shows quantification of expression of CD163 (*n* = 66) or NOS2 (*n* = 98) in the outer/host cells of CICs identified. Scale bar: 15 μm.

Analysis of the CICs in stomach tumors identified four subtypes of structures, with two of them CD68^−^ in outer cells, which are usually E-cadherin^+^ tumor cells; and two are CD68^+^ in outer cells, suggesting macrophage-mediated engulfment. These four subtypes are indicated as: 1) CD45^−^/CD68^−^, with CD45^−^ cells in CD68^−^ tumor cells, suggesting homotypic CICs between tumor cells that are positive in E-cadherin, characterized by one cell surrounded by another with crescent nucleus, the intercellular boundary is positive in E-cadherin that usually appears as round circle (Figure 4A); 2) CD45^+^/CD68^−^, with CD45^+^ cells in CD68^−^ tumor cells, referring to tumor cells engulfing lymphocytes (Figure 4B); 3) CD45^−^/CD68^+^, with CD45^−^ cells in CD68^+^ cells, for tumor cells engulfed by macrophages (Figure 4C); 4) CD45^+^/CD68^+^, with CD45^+^ cells in CD68^+^ tumor cells, for macrophages engulfing lymphocytes (Figure 4D). It should be noted that CD45^+^ cells, no matter engulfed by tumor cells or macrophages, usually displayed weak staining in DAPI, suggesting being digested in the lysosome, which is consistent with the finding that lymphocytes are usually short-lived once being engulfed [13]. As we know that macrophages are usually polarized in human tumors, we therefore detected the co-expression of CD68 with NOS2 (the marker for M1 macrophage) or CD163 (the marker for M2 macrophage). As shown in Figure 5, out of 66 CICs, we got 3 CICs (5%) that are double positive in both CD68 and CD163, meanwhile, we didn't see one CICs that is double positive in CD68 and NOS2 from 98 CICs, suggesting that the M2 macrophage could participate in CICs formation in human tumors.

### Profiles of CICs subtypes in human tumors

**Figure 6:**
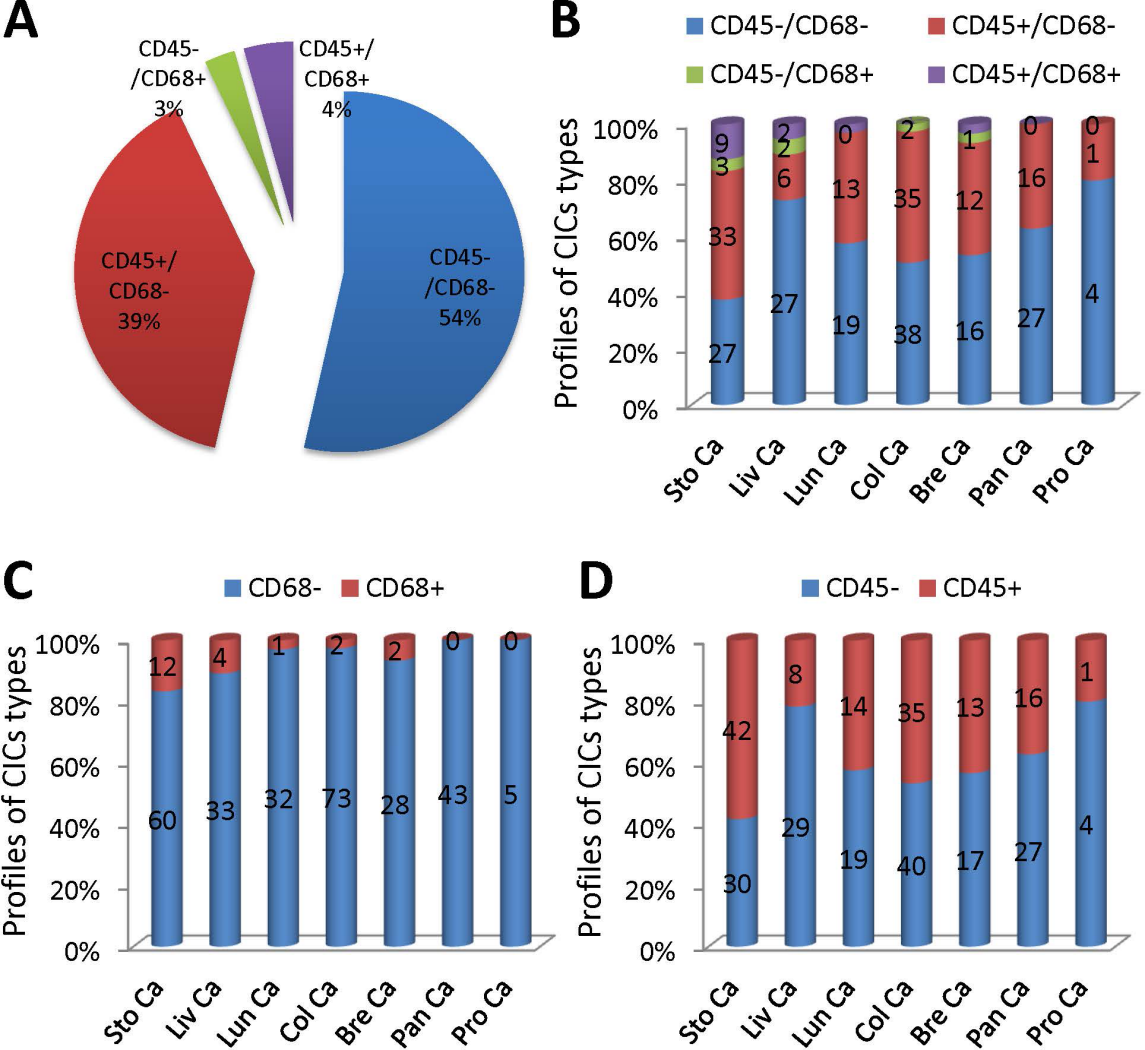
Analysis of CICs subtypes in human tumors **A.** Pie graph shows the distribution of four CICs subtypes in all CICs indentified from various tumor TMAs. **B.** The distribution of four CICs subtypes in CICs identified in individual tumor TMA. **C.** The distribution of CD68^+^ CICs and CD68^−^ CICs identified in individual tumor TMA. **D.** The distribution of CD45^+^ CICs and CD45^−^ CICs identified in individual tumor TMA. Sto Ca: stomach carcinoma; Liv Ca: liver carcinoma; Lun Ca: lung carcinoma; Col Ca: colon carcinoma; Bre Ca: breast carcinoma; Pan Ca: pancreas carcinoma; Pro Ca: prostate carcinoma. *n* = 295.

Quantification of all CICs in the tumor samples examined revealed that tumor cell-mediated engulfment (CD68^−^) constituted the majority of the structures identified (93%), only a small portion of them were mediated by macrophages (CD68^+^) (Figure 6A). For cells that were engulfed, the number of tumor cells (CD45^−^) was comparable to that of lymphocytes (CD45^+^) with tumor cells a bit more (Figure 6A). Homotypic CICs between tumor cells (CD45^−^/CD68^−^) (54%) were similar in number to heterotypic CICs (CD45^+^/CD68^−^, CD45^−^/CD68^+^, CD45^+^/CD68^+^) (Figure 6A). The distribution pattern of overall CICs generally represented that of individual tumor type. For example, the numbers of homotypic CICs and heterotypic CICs were similar in tumors from colon, lung and breast gland (Figure 6B); CD68^+^ macrophage-mediated CICs were rare in all tumors of each type, with no CD68^+^ CICs identified in tumors from pancreas and prostate (Figure 6C). Nevertheless, each tumor type harbored its own unique CICs pattern. For instance, homotypic CICs were less than heterotypic CICs in stomach tumors, which might be due to some extents of inflammation these tumors were undergoing (Figure 6B and 6D). Not all CICs subtypes could be identified in a specified tumor type (Figure 6B). Therefore, careful CICs subtyping is warranted for each tumor type and sample as well.

## DISCUSSION

Thanks to mechanistic studies on several models, including entosis [8], emperitosis [13], heterotypic cell cannibalism (HeCC) [12], homotypic cell cannibalism (HoCC) [10] and suicidal emperipolesis (SE) [26] and the like, CICs formation was found a unique way to mediate cell death. Moreover, the context-dependent feature fits CICs-mediated death well as a form of cell competition in mammalian cells [16, 17]. These landmark advances made the old cell-in-cell phenomenon one of the fundamental processes in homeostasis maintenance of our body, aberration of which would result in pathological conditions like tumors and autoimmune diseases [18, 26].

Long before the mechanistic studies, pathologists had attempted to associate CICs in clinical specimens with clinical features. For examples, Kojima *et al* examined voided urine of a cohort of 252 bladder cancer patients, and found that patients with CICs showed significantly higher rate of progression than those without CICs, thus proposed CICs an independent factor for the prediction of progression [27]; Gupta *et al* found the presence of CICs could differentiate benign and malignant tumors [2], on which, however, some others provided contradict evidences [25]. The obvious controversy may come from different samples used or suggest that factors, such as CICs subtypes in addition to CICs frequency, might be accountable. Consistent with this, homotypic and heterotypic CICs were found being able to impart different effects on tumor growth [8, 10, 12, 16]. However, CICs were rarely subtyped in most of the clinical researches on CICs, which promotes us to analyze the cell types involved in CICs in human tumors.

In this work, we identified four CICs subtypes in various human tumors (Figures 4 and 6) based on the cell pairs forming CICs. Nevertheless, it's conceivable that other CICs types might exist, such as tumor cells engulfing macrophages, which might be identified in theory if more samples were examined. An interesting finding is that the macrophages involving in CICs formation was positive in CD163 (Figure 5), a marker for M2 macrophage, suggesting that M2 macrophages might influence human tumors via a novel mechanism, which warrants further investigation. Also as for those CICs with CD45^+^ leukocytes internalized, it will be interesting to further characterize what kind of leukocytes were internalized since a range of them had been reported before, including NK cells, T and B cells, and neutrophils etc. Due to limited samples for each tumor type, we didn't perform an association analysis between CICs, frequency or subtype, and clinical features, however, valuable information would be expected in future if large number of tissues were examined.

To identify CICs in tissues, it's prerequisite to indicate cell boundary. Here we use E-cadherin to label cell membrane since most of the tumors we examined are epithelium origin. However, E-cadherin did not always label tumor cells because some tumor cells lost expression of this gene, in which case, some other molecules are needed. Given the complex gene expression patterns in different tumors, this issue may be solved in a context-dependent way, that is, identifying an ideal marker for each type of tumors. Another important issue on scoring CICs is the inner cell viability, although CICs specifically refer to those with viable cell engulfment, they will eventually result in inner cell death, which made them morphologically indistinguishable to phagocytosis-mediated structures *in situ*. Considering this scenario, we here count CICs solely based on morphology irrespective of their forming mechanisms, thus, all structures with one or more cells inside of another were counted no matter the inner cells were morphologically viable or dead. And we also recommend this scoring standard for similar investigation in future.

Researches on a specified field rely on proper models and feasible methods. We previously reported the method to quantify homotypic CICs by immunostaining and microscopic observation [28], which had been widely used for CICs studies. And lately, we developed a high-through method to quantify heterotypic CICs based on flow cytometry analysis, which turned out to be quite efficient to enrich CICs with purity higher than 90% [29]. In this work, we described a method, named EML method based on cell types examined, to analyze CICs in tissues. Altogether we would anticipate these methods would speed up future researches on CICs if adopted for application.

## MATERIALS AND METHODS

### Human tumor tissue microarray and antibodies

Human tumor tissue microarray (TMA) slides were purchased from *Biomax, Inc*. The test arrays for tumors from seven different tissues as listed below (Table 1) were used for immunostaining. Each array contains 24 cores representing for 12 different samples (cases), duplicate cores per case, and is divided into two identical 12 core arrays, among which 10 cores are from tumors and 2 from normal tissues. Core diameter is 1.5 mm, thickness is 5 um. For detail information of each sample plotted on the slides, please visit the website of *Biomax* by the address of http://www.biomax.us/tissue-arrays.

**Table 1:** Information of each TMA test slide used in this study

Code	Tissue	Composition
T086d	Breast	4 cases of breast invasive ductal carcinoma, 2 each of breast medullary carcinoma and mucinous carcinoma, 1 each of breast neuroendocrine carcinoma and invasive lobular carcinoma, plus 2 breast tissues
T032a	Liver	5 cases of liver hepatocellular carcinoma, 2 liver cholangiocellular carcinoma, 1 each of liver clear cell carcinoma, malignant fibrohistiocytoma and angiosarcoma, plus 2 normal liver tissues
T045d	Lung	2 cases of lung adenocarcinoma, 1 each of lung papillary adenocarcinoma, squamous cell carcinoma, adenosquamous carcinoma, bronchioloalveolar carcinoma, large cell carcinoma, small cell undifferentiated carcinoma, atypical carcinoid and carcinoid, plus 2 normal lung tissues
T054b	Colon	7 cases of adenocarcinoma, 1 each of adenosquamous carcinoma, neuroendocrine carcinoma and leiomyosarcoma and 2 normal colon tissues
T0142a	Pancreas	2 cases of pancreas duct adenocarcinoma, 2 each of pancreas adenosquamous carcinoma and neuroendocrine carcinoma, 1 each of pancreas solid pseudopapillary tumor, solid-cystic pseudopapillary tumor, leiomyosarcoma and islet cell tumor, plus 2 normal pancreatic tissues
T0195b	Prostate	9 cases of prostate adenocarcinoma and 1 prostate low grade malignant leiomyosarcoma, plus 2 normal prostate tissues
T012a	Stomach	10 cases of stomach tumor and 2 normal tissues

### Immunostaining of TMA

Slides were routinely de-paraffinized with Xylene-Ethonal method following baked in 65°C for 1.5 hour. Antigen retrieval was performed in citrate acid buffer by microwaving method for 15 min after boiling, followed by 1 hour blocking in 5% BSA in TBS. Samples were first stained with antibody against CD45 (mouse mAb from *Boster*, BM0091) at dilution of 1:400 by Opal Multiplex tissue staining kit (*Perkin Elmer*, NEL791001KT) according to the standard protocol provided, CD45 molecules were eventually labeled with Cyanine 5 fluorophore. Slides were then incubated with mixed antibodies against E-cadherin (mouse mAb from *BD Biosciences*, 610181) and CD68 (rabbit pAb from *Proteintech*, 25747–1-AP), followed by secondary antibodies of Alexa Fluor 568 anti-rabbit antibody (*Invitrogen*, A11036) and Alexa Fluor 488 anti-mouse antibody (*Invitrogen*, A11029). For macrophage subtyping, tissues were first stained with E-cadherin (mouse mAb from *BD Biosciences*, 610181) at dilution of 1:1000 by Opal protocol, and then with mixed antibodies against CD68 (mouse mAb from *Santa Cruz*, sc-20060) and NOS2 (rabbit pAb from *Boster*, BA0362) or CD163 (rabbit pAb from *Boster*, BA13856). Samples were also labeled with single fluorophore to acquire spectral signatures. All slides were counterstained with DAPI to show nuclei and mounted with Antifade reagent (*Invitrogen*, Carlsbad, CA) and cover slips followed by sealing with nail oil.

### Multispectral imaging and analysis

Multispectral images were taken with TMA modules of Vectra® Automated Imaging System (*Perkin Elmer*) by 20x objective lens. Nuance system (*Perkin Elmer*) was used to build libraries of each spectrum (DAPI, 488, 568 and Cy5) and unmix multispectral images with high contrast and accuracy. InForm automated image analysis software package (*Perkin Elmer*) was used for batch analysis of multispectral images based on specified algorithms.

### CICs quantification in tumor samples

Cellular structure where one or more cells morphologically fully enclosed inside of another cell with crescent nucleus was scored as CICs. Since the CICs could result in inner cell death, therefore, we scored all structures displaying CICs morphology irrespective of the status (dead or live) of inner cells. Cell boundary could be told by E-cadherin, which labels cell membrane, and CD68, which labels cell body. For efficient quantification, CICs were usually first screened in a composite image of four fluorescent channels and then confirmed in unmixed channels.

### Confocal microscopy

Images of high resolution were taken by Olympus FV1000 line scanning confocal microscope at four channels (DAPI, 488, 568 and Cy5), and analyzed by FV10-ASW 4.0 Viewer software.
